# Dose response relationship between D-dimer level and mortality in critically ill COVID-19 patients: a retrospective observational study

**DOI:** 10.12688/f1000research.108972.1

**Published:** 2022-03-03

**Authors:** Dita Aditianingsih, Ratna Farida Soenarto, Artheta Mutiara Puiantana, Raymond Pranata, Michael Anthonius Lim, Putu Angga Risky Raharja, Ponco Birowo, Markus Meyer

**Affiliations:** 1 Division of Critical Care, Universitas Indonesia Hospita, Depok, Jawa Barat, Indonesia; 2 Department of Anesthesia and Intensive Care, Dr. Cipto Mangunkusumo Hospital – Universitas Indonesia Hospital, Jakarta, DKI Jakarta, Indonesia; 3 Faculty of Medicine, Pelita Harapan University, Tangerang, Banten, Indonesia; 4 Department of Urology, Dr. Cipto Mangunkusumo Hospital – Universitas Indonesia Hospital, Jakarta, DKI Jakarta, Indonesia; 5 Faculty of Medicine, Universitas Indonesia, Jakarta, DKI Jakarta, Indonesia

**Keywords:** COVID-19; critically ill; D-dimer; dose-response relationship; mortality

## Abstract

Background: Coronavirus disease 2019 (COVID-19) is a global pandemic. Coagulopathy is one of the most common complications characterized by increased D-dimer level. We aimed to investigate the dose-response relationship between elevated D-dimer level and mortality in critically ill COVID-19 patients.

Methods: This was a retrospective observational study in 259 critically ill COVID-19 patients requiring intensive care unit admission between March and December 2020. We compared the mortality rate between patients with and without elevated D-dimer. Receiver operating characteristic (ROC) curve analysis, Fagan’s nomogram, and dose-response relationship were performed to determine the association between D-dimer level and mortality.

Results: Overall mortality rate was 40.9% (106 patients). Median D-dimer level was higher in non-survivor group (10,170 ng/mL vs 4,050 ng/mL, p=0.028). The association remained significant after multivariate logistic regression analysis (p=0.046). The optimal cut-off for D-dimer level to predict mortality from ROC curve analysis was 9,020 ng/mL (OR (odds ratio) 3.73 [95% CI (confidence interval) 1.91 – 7.28], p<0.001). D-dimer level >9,020 ng/mL confers 67% posterior probability of mortality and D-dimer level <9,020 ng/mL had 35% probability of mortality.

Conclusions: There was a non-linear dose-response relationship between D-dimer level and mortality with P _nonlinearity_ of 0.004. D-dimer level was associated with mortality in critically ill COVID-19 patients in the non-linear dose-response relationship.

## Introduction

Currently, the coronavirus disease 2019 (COVID-19) is one of the most common diseases affecting society globally, causing a sizeable number of deaths as of 2022. ^1^ Although the majority of patients had only mild-moderate clinical manifestations, a small but significant proportion developed life-threatening complications, for example, multiple organ failure. ^2^^–^^4^ This is especially true in patients with comorbidities, such as diabetes mellitus (DM), chronic kidney disease (CKD), and cerebrovascular and cardiovascular diseases, that are associated with increased severity and mortality from COVID-19. ^5^^–^^11^ The cases of COVID-19 remain high and threaten to overwhelm the healthcare system, therefore risk stratification is required for prudent allocation of resources. Several biomarkers have been evaluated or repurposed to attain this objective. ^12^^–^^14^

The importance of simple laboratory values, such as complete blood count, coagulation parameters, and inflammatory biomarkers, cannot be underestimated during the ongoing COVID-19 pandemic. ^15^^–^^18^ These values can help predict the severity of the disease course and often indicate the need for intensive care unit (ICU) admission or mechanical ventilation. Coagulopathy is among the most frequently found complications, which is characterized by the elevation of D-dimer level and changes in fibrinogen levels and platelet counts. ^19^^,^^20^ Although increased D-dimer levels have been consistently observed in severely and critically ill patients, the optimal cut-off level and prognostic values remain unknown. ^21^ This study aimed to investigate the dose-response relationship between elevated D-dimer level and mortality in critically ill COVID-19 patients.

## Methods

### Ethics

This study was conducted in accordance with the ethical standards of the Helsinki Declaration. The Ethics Committee of the Faculty of Medicine, Universitas Indonesia (date 15.06.20, No. 0593/UN2.F1/ETIK/PPM.00.02/2020) approved the study protocol. Informed consent was not obtained because the study was retrospective observational in nature.

### Study design

This study was a retrospective observational study in Cipto Mangunkusumo Hospital and Universitas Indonesia Hospital, Indonesia. There were 261 critically ill COVID-19 patients requiring intensive care unit (ICU) admission between March and December 2020. COVID-19 diagnosis was based on a reverse transcriptase-polymerase chain reaction (RT-PCR) examination. We did consecutive enrollment to address any potential sources of bias. Data on the patients’ baseline clinical and laboratory characteristics were collected in a list form from the medical records in December 2020 and then analyzed using IBM SPSS Statistics version 25. We used a multivariate analysis to adjust the possible effect of confounders.

### Outcome

The outcome of this study was mortality, defined as clinically validated death/non-survivor. The outcome was ascertained from the medical record and confirmed by the death certificate. An independent investigator of the data collection process and patient care performed the statistical analysis. We compared the mortality rate between the patients with elevated D-dimer level and those without.

### Statistical analysis

We performed the statistical analysis using SPSS 25.0 (Armonk, US) and STATA 14.0 (College Station, TX, US). We tested continuous data for normal distribution; t-test was used for normally distributed data, and Mann-Whitney test was used for abnormally distributed data. Chi-square test or Fischer-Exact test was performed for categorical variables. Normally distributed continuous data were presented as mean and standard deviation (SD); while abnormally distributed continuous data were reported as the median and interquartile range (IQR). Bivariate analysis was performed to evaluate variables. Continuous variables were compared using either independent T or Mann-Whitney U test. Categorical variables were tested by using chi-square test. Significant variables (P<0.05) were included in the logistic regression analysis. ROC curve analysis was performed to determine the optimal cut-off points for the D-dimer level. The sensitivity, specificity, positive likelihood ratio (LR+), negative likelihood ratio (LR-), and area under the curve (AUC) were calculated. Fagan’s nomogram was plotted to determine the posterior probability of mortality for the cut-off points determined by ROC curve analysis. The dose-response relationship between D-dimer level and mortality in the patients was explored and reported in the form of a restricted cubic spline.

## Results

This study enrolled 261 critically ill COVID-19 patients between March 2020 and December 2020 in this study. Two patients were excluded due to missing data, so 259 patients who met the inclusion criteria were included in the analysis. The mortality rate was 40.9% (106 patients). Demographic characteristics, preoperative laboratory parameters, and comorbidities of the patients were presented in Table 1. Patients in the non-survivor group were older than the survivor group. The mean ages of the non-survivor group and survivor group are 56.04 years and 46.73 years, respectively (p<0.001). There was no significant difference in gender distribution between groups. Non-survivors were more likely to have higher D-dimer levels, leukocyte levels, C-reactive protein levels, and lower thrombocyte levels. Comorbidities such as hypertension, coronary artery disease, and chronic kidney disease were more frequently found in the non-survivor group.

**Table 1.** Demographic, laboratory parameters, and comorbidities between survivor and non-survivor group.

Variables	Non-survivor group (N=106)	Survivor group (N=153)	P value
Age (years)	56.04±15.12	46.73±15.66	**<0.001**
Gender			0.591
Male	72 (67.9%)	99 (64.7%)	
Female	34 (32.1%)	54 (35.3%)	
Laboratory parameters			
D-dimer level (ng/mL)	10,170 (3007.5 – 21,497.5)	4,050 (1,740 – 8,210)	**0.028**
Hemoglobin (g/dL)	11.32±2.89	11.82±2.74	0.154
Leukocyte (×10 ^3^ cells/μL)	13.66 (8.50 – 18.46)	10.59 (7.05 – 15.92)	**0.013**
Thrombocyte (×10 ^3^ cells/μL)	235.5 (174.5 – 335.8)	308.0 (222.0 – 415.0)	**<0.001**
C-reactive protein (mg/L)	182.0 (112.3 – 305.7)	140.1 (65.5 – 195.9)	**<0.001**
Procalcitonin (mg/dL)	3.38 (0.43 – 20.15)	0.89 (0.24 – 4.80)	0.718
Comorbidities			
Hypertension	57 (53.8%)	60 (39.2%)	**0.021**
Coronary artery disease	28 (26.4%)	21 (13.7%)	**0.010**
Diabetes mellitus	32 (30.2%)	38 (24.8%)	0.340
Chronic kidney disease	29 (27.4%)	14 (9.2%)	**<0.001**

### D-dimer level and mortality

Median D-dimer level was higher in the non-survivor group (10,170 ng/mL vs 4,050 ng/mL, p=0.028). This association remained significant after logistic regression multivariate analysis (p=0.046), as shown in Table 2.

**Table 2.** Multivariate logistic regression analysis of significant variables.

Variables	P value
Age	**<0.001**
D-dimer level	**0.046**
Leukocyte	0.189
Thrombocyte	0.070
C-reactive protein	**0.014**
Hypertension	0.787
Coronary artery disease	0.177
Chronic kidney disease	0.315

**Figure 1.**
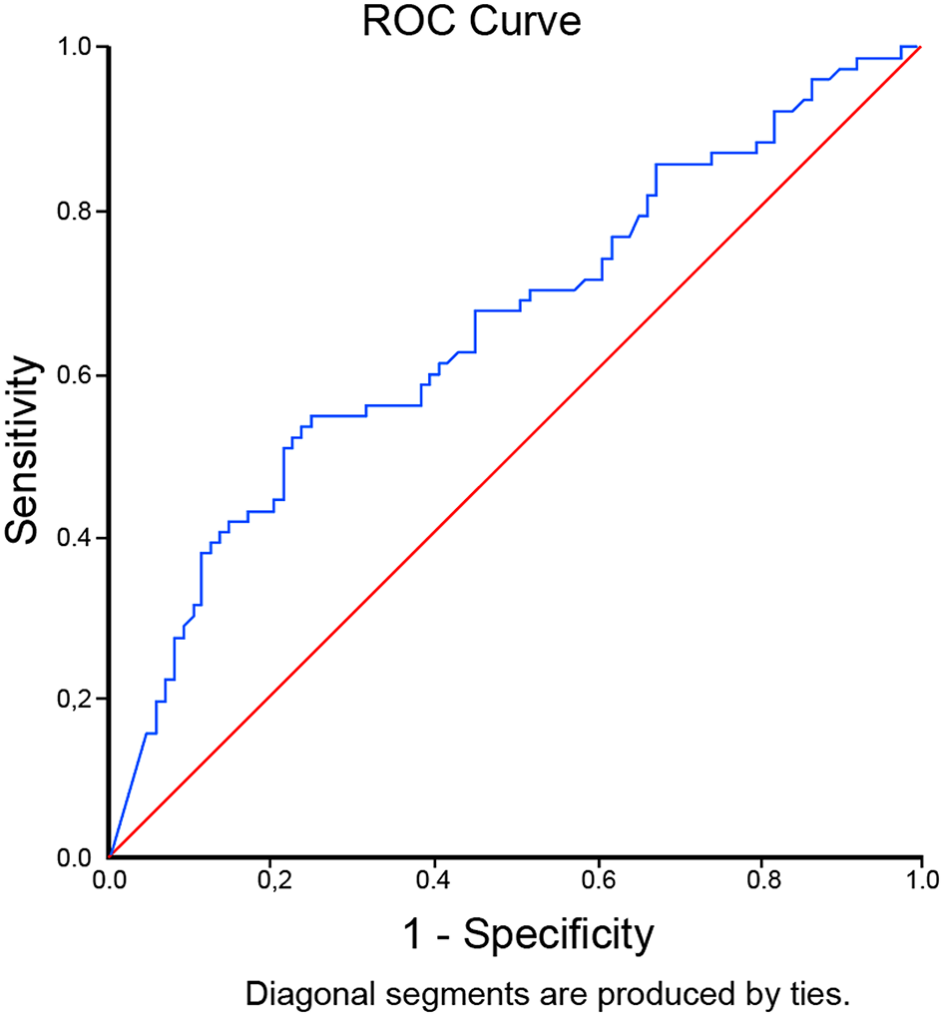
ROC curve analysis of D-dimer levels with cut off of 9,020 ng/mL to predict mortality in critically ill COVID-19 patients.

**Figure 2.**
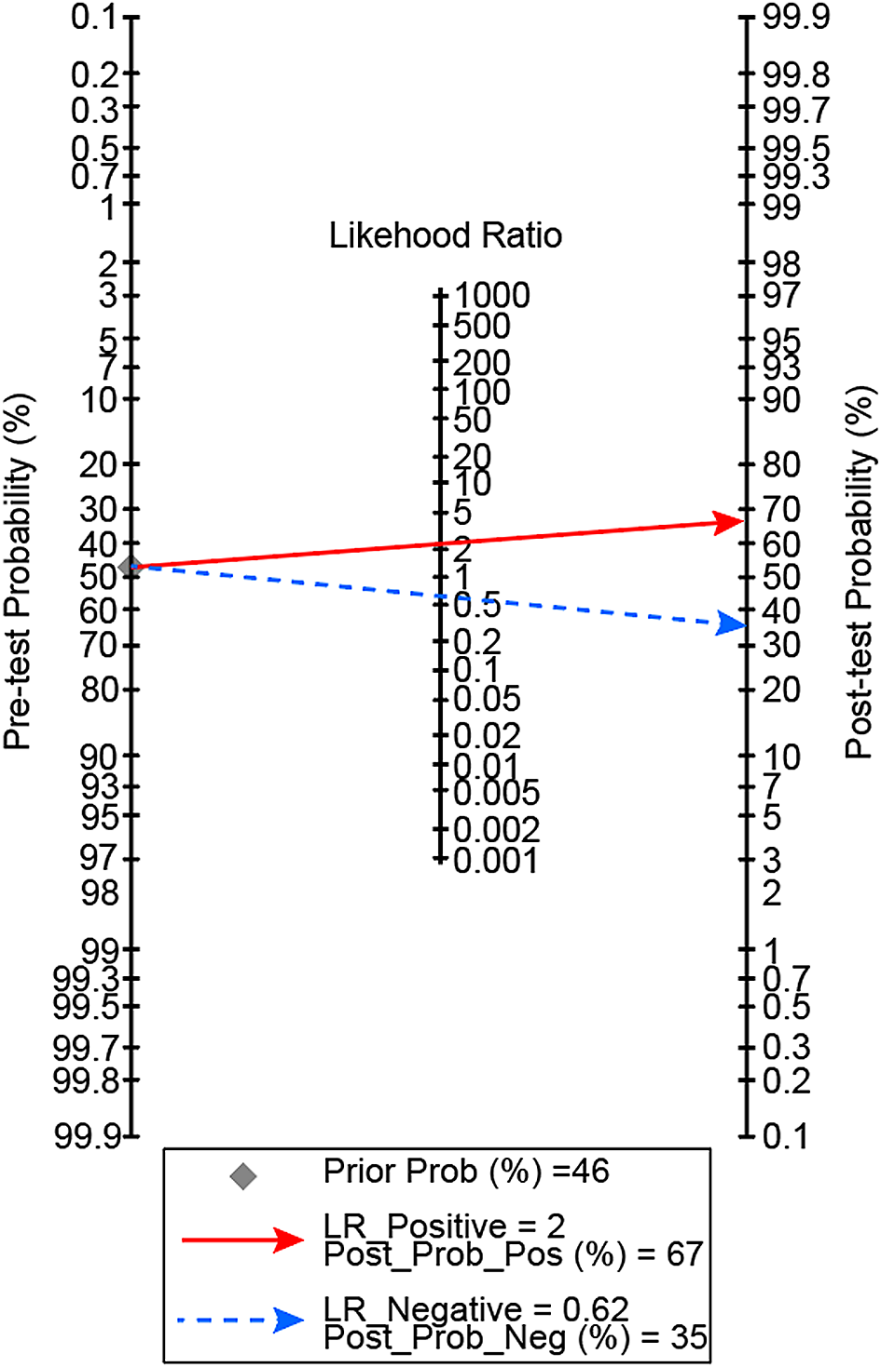
Posterior probability of mortality with D-dimer level cut off of 9,020 ng/mL.

**Figure 3.**
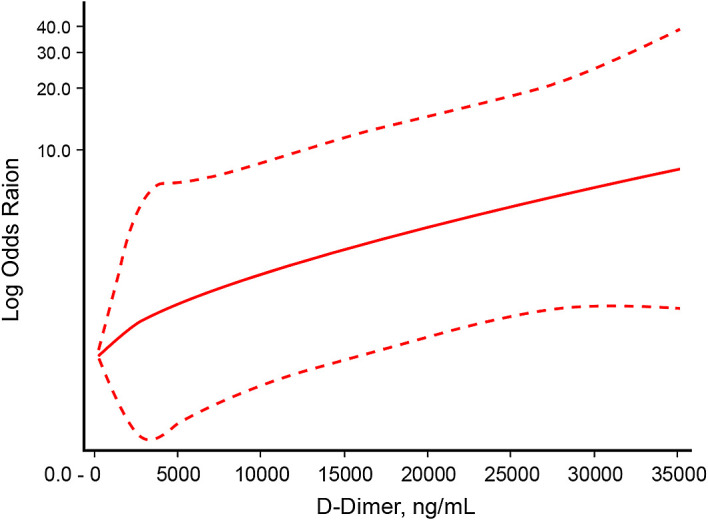
Non-linear dose-response relationship between D-dimer level and mortality.

The optimal cut-off level for D-dimer to predict mortality in critically ill COVID-19 patients was determined to be 9,020 ng/mL (OR 3.73 [95% CI 1.91 – 7.28], p<0.001) through ROC curve analysis. It was associated with 52% sensitivity, 78% specificity, LR+2.31, and LR- 0.62. The AUC was 0.657 (95% CI 0.573-0.740), p=0.001 [ Figure 1]. In our patients, a D-dimer level of >9,020 ng/mL gave 67% posterior probability of mortality, and a D-dimer level of <9,020 ng/mL had 35% probability of mortality [ Figure 2]. There was a non-linear dose-response relationship between D-dimer level and mortality with P _nonlinearity_=0.004 [ Figure 3].

## Discussion

This study showed that D-dimer level has a non-linear dose-relationship with mortality in critically ill COVID-19 patients. The optimal D-dimer level cut-off point was 9,020 ng/mL. An elevation beyond the cut-off point confers to 67% posterior probability of mortality, and D-dimer level of <9,020 ng/mL had 35% probability of mortality.

D-dimer is a fragment produced when plasmin cleaves fibrin during clot breakdown. In clinical practice, D-dimer assays are frequently used to exclude a diagnosis of deep vein thrombosis (DVT) and pulmonary embolism (PE). Abnormal coagulation function, specifically blood clotting, is often indicated by an elevation in D-dimer level, reflecting a severe viral infection. Elevated D-dimer level is associated with COVID-19 progression as well as a greater death rate from community-acquired pneumonia (CAP). ^17^^,^^22^ For this reason, testing D-dimer level on admission could be useful to stratify patients with COVID-19 early and to determine the treatment plan. A high on-admission D-dimer level might suggest a prothrombotic state, increased fibrinolysis, bleeding, and thrombotic events, and indicate cytokine storm, tissue damage, or impending sepsis. ^17^^,^^23^

Elevated D-dimer level has been shown to be associated with mortality in COVID-19 patients. ^17^ This study showed that D-dimer level can be used for prognostication in critically ill COVID-19 patients and also showed the non-linear dose-response relationship of D-dimer level and mortality. Elevation of inflammatory cytokines and biomarkers such as interleukin (IL)-2, IL-6, IL-7, granulocyte-colony stimulating factor, macrophage inflammatory protein 1-α, tumor necrosis factor-α, C-reactive protein (CRP), ferritin, procalcitonin (PCT), and D-dimer level were found in the systemic hyperinflammation phase. ^17^^,^^24^ Excessive hyperinflammation consequently leads to multi-organ failure ^24^^–^^26^; therefore D-dimer level may reflect the severity of inflammation. Additionally, SARS-CoV-2 can induce hypercoagulability, ^27^^,^^28^ causing thrombosis in cardiovascular systems as well as myocardial injuries. ^29^ Coagulopathy and cardiac injury results in higher mortality in patients with COVID-19, ^3^^,^^26^^,^^30^^,^^31^ which may explain the relationship between D-dimer level and mortality in COVID-19 patients.

There is a crosstalk between blood coagulation and inflammation. The release of pro-inflammatory cytokines results in the upregulation of tissue factor (TF) expression on the endothelial cells and monocytes surfaces, which further promotes procoagulant activity. ^32^ Moreover, hypoxia in COVID-19, CAP, or other illnesses may stimulate thrombosis by upregulating the hypoxia-inducible transcription factors which modulate the expression of various coagulation and fibrinolytic factors such as TF, tissue factor pathway inhibitor, and plasminogen activator inhibitor-1 (PAI-1). ^33^ In addition, neutrophil extracellular traps, in which neutrophil infiltrates lung capillaries and cause fibrin deposition and acute inflammation, may exacerbate COVID-19 progression by causing organ damage and promoting thrombosis. ^34^

International Society of Thrombosis and Haemostasis (ISTH) guideline states that an elevated D-dimer levels indicates increased thrombin production. COVID-19 patients with markedly elevated D-dimer level may require hospitalization despite the severity of clinical presentation. ^17^^,^^35^ Prophylactic anticoagulant is recommended in hospitalized patients with COVID-19 in the absence of contraindication.

A limitation of this study was the small sample size of patients from two centers that needed further external validation to support the clinical practice. This research began at the beginning of the pandemic, hence there were adjustments to the system for recording and storing medical records for COVID-19 patients. Secondary data was taken from the medical records, which had a risk of selection, recall, or misclassification bias. This study is a cross-sectional descriptive study, which was influenced by several data biases such as data availability, different follow-up examination, and different given therapies because several patients underwent another different study as this study was held.

## Conclusion

D-dimer level was associated with mortality in critically ill COVID-19 patients in the non-linear dose-response relationship.

## Data availability

### Underlying data

figshare: Data Set (D-Dimer).xlsx. https://doi.org/10.6084/m9.figshare.17125520.v5

This project contains the following files:

- Data Set (D-Dimer) – Outcome.xlsx (Outcome of the subjects whether died or stepped down from ICU)
- Data Set (D-Dimer) – Characteristics.xlsx (Characteristics of the subjects, such as gender, age, body weight, comorbidities, and lab result)
- Data Set (D-Dimer) – Lab.xlsx (Lab result from each subject)

Data are available under the terms of the Creative Commons Attribution 4.0 International license (CC-BY 4.0).
