# Supplementary material for: Dose response relationship between D-dimer level and mortality in critically ill COVID-19 patients: a retrospective observational study
Source: F1000Res. 2023 Jan 10;11:269. Originally published 2022 Mar 3. [Version 2] doi: 10.12688/f1000research.108972.2 (PMC11043662; doi:10.12688/f1000research.108972.2)
Supplement: Peer review history

# Reviewer report: Reviewer response for version 2

**DOI:** 10.5256/f1000research.139993.r169657

**Published:** 2024-04-24

**Authors:** Luca Spiezia

**Affiliations:** 1 Padua University, Padua, Italy

I read with interest the paper written by Ratna Farida Soenarto et al. that describes the association between D-dimer plasma levels and mortality in a group of n. 259 critically ill COVID-19 patients admitted to Intensive Care Unit (ICU) between March and December 2020. The authors found a significant association between D-dimer levels and mortality.

 My comments:

 1. I think the authors have looked at in-hospital mortality but it's not written anywhere. Please specify throughout the text.

 2. When was the dimer sample taken?

 the type of test used to determine d-dimer value must be specified so that data from this study can be compared with data from other studies

 3. There are no inclusion and exclusion criteria.

 4. It’s necessary to stratify cases and controls according to the severity of the infection (using for example the SOFA score) and it’s also necessary to add to the multivariate model the SOFA score.

 5. The sentence “Coagulopathy is one of the most common complications characterized by increased D-dimer level” reported in the abstract is reductive and misleading. Several papers have been published in the literature describing the coagulation alterations in patients with Covid-19 related infection. Please delete/rewrite.

 6. Both in the “Introduction” section and in the “Discussion” section the association among hypercoagulability (i.e. Spiezia L et al. Thromb Haemost. 2020 Jun;120(6):998-1000; Ranucci M et al. J Thromb Haemost. 2020 Jul;18(7):1747-1751; Pianigada M et al. J Thromb Haemost. 2020 Jul;18(7):1738-1742) and venous thromboembolism (i.e. Klok FA et al, Thromb Res. 2020 Jul;191:145-147, Middeldorp S et al, J Thromb Haemost. 2020 Aug;18(8):1995-2002, Lodigiani C et al, Thromb Res. 2020 Jul;191:9-14, Avruscio G et al. Clin Transl Sci. 2020 Nov;13(6):1108-1114) and mortality needs to be considered much more in detail.

Is the work clearly and accurately presented and does it cite the current literature?

Partly

If applicable, is the statistical analysis and its interpretation appropriate?

Yes

Are all the source data underlying the results available to ensure full reproducibility?

Yes

Is the study design appropriate and is the work technically sound?

Yes

Are the conclusions drawn adequately supported by the results?

Yes

Are sufficient details of methods and analysis provided to allow replication by others?

Partly

Reviewer Expertise:

Coagulation disorders and venous thromboembolism

I confirm that I have read this submission and believe that I have an appropriate level of expertise to confirm that it is of an acceptable scientific standard, however I have significant reservations, as outlined above.

# Reviewer report: Reviewer response for version 2

**DOI:** 10.5256/f1000research.139993.r159592

**Published:** 2023-01-23

**Authors:** Martin Westphal

**Affiliations:** 1 University Hospital of Munster, Germany; Fresenius Kabi Deutschland GmbH, Bad Homburg, Germany

The authors took care of the comments raised by the reviewers and revised the manuscript appropriately.

Is the work clearly and accurately presented and does it cite the current literature?

Yes

If applicable, is the statistical analysis and its interpretation appropriate?

Yes

Are all the source data underlying the results available to ensure full reproducibility?

Yes

Is the study design appropriate and is the work technically sound?

Yes

Are the conclusions drawn adequately supported by the results?

Yes

Are sufficient details of methods and analysis provided to allow replication by others?

Yes

Reviewer Expertise:

Anesthesiology and intensive care

I confirm that I have read this submission and believe that I have an appropriate level of expertise to confirm that it is of an acceptable scientific standard.

# Reviewer report: Reviewer response for version 1

**DOI:** 10.5256/f1000research.120419.r147444

**Published:** 2022-09-07

**Authors:** Reza Zolfaghari Emameh

**Affiliations:** 1 NIGEB, Tehran, Iran

The manuscript entitled “Dose response relationship between D-dimer level and mortality in critically ill COVID-19 patients: a retrospective observational study” was reviewed carefully. The language is acceptable and the subject has great importance, although it is not the first report on the association of D-dimer high concentration with high mortality rate. Please see the following publication: https://www.scielo.br/j/clin/a/w3HnMz3KjhCfSJWVhfKMjRS/?format=pdf&lang=en.

- Please state briefly about the literature on other factors having an association with high mortality rate or prepare a brief table with proper citations in the “Introduction”, such as PMID: 32572334 ^1^, PMID: 32754004 ^2^, PMID: 33407800 ^3^, PMID: 33083287 ^4^, and PMID: 36043922 ^5^.
- Table 1: Why the diabetes survivor group has a higher percentage than the non-survivor group? I think diabetes patients are in the high-risk group.
- Table 1: Why the thrombocyte percentage is higher in the survivor group than the non-survivor group? High thrombocyte count is one of the main reasons for VTE in COVID-19.

Therefore, after performing the modifications and due to the decision of the respected editor, this manuscript can be indexed.

Is the work clearly and accurately presented and does it cite the current literature?

Partly

If applicable, is the statistical analysis and its interpretation appropriate?

Yes

Are all the source data underlying the results available to ensure full reproducibility?

Yes

Is the study design appropriate and is the work technically sound?

Yes

Are the conclusions drawn adequately supported by the results?

Yes

Are sufficient details of methods and analysis provided to allow replication by others?

Partly

Reviewer Expertise:

Biotechnology, Bioinformatics, Carbonic anhydrase, SARS-CoV-2, COVID-19

I confirm that I have read this submission and believe that I have an appropriate level of expertise to confirm that it is of an acceptable scientific standard, however I have significant reservations, as outlined above.

## References

[1] : Application of System Biology to Explore the Association of Neprilysin, Angiotensin-Converting Enzyme 2 (ACE2), and Carbonic Anhydrase (CA) in Pathogenesis of SARS-CoV-2. *Biol Proced Online* .2020;22: 10.1186/s12575-020-00124-6 11 10.1186/s12575-020-00124-6 32572334 PMC7302923

[2] : Expansion of Single Cell Transcriptomics Data of SARS-CoV Infection in Human Bronchial Epithelial Cells to COVID-19. *Biol Proced Online* .2020;22: 10.1186/s12575-020-00127-3 16 10.1186/s12575-020-00127-3 32754004 PMC7377208

[3] : Identification and characterization of a silent mutation in RNA binding domain of N protein coding gene from SARS-CoV-2. *BMC Res Notes* .2021;14(1) : 10.1186/s13104-020-05439-x 10 10.1186/s13104-020-05439-x 33407800 PMC7787625

[4] : Factors Associated with Mortality in COVID-19 Patients: A Systematic Review and Meta-Analysis. *Iran J Public Health* .2020;49(7) : 10.18502/ijph.v49i7.3574 1211-1221 10.18502/ijph.v49i7.3574 33083287 PMC7548487

[5] : Factors affecting mortality in COVID-19 patients in intensive care. *Ulus Travma Acil Cerrahi Derg* .2022;28(9) : 10.14744/tjtes.2021.37468 1229-1237 10.14744/tjtes.2021.37468 36043922 PMC10315946

# Response

**Published:** 2022-10-18

**Authors:** Dita Aditianingsih

**Affiliations:** Dr. Cipto Mangunkusumo Hospital, Indonesia

First, thank you for the review.

1. We will add the article as you suggested.
2. ​​​​​​We agree with the reviewer that diabetic patients have a higher risk. Although the diabetes survivor was higher, there was no significant difference in our patient for diabetes comorbidities (P =0.340), and it could be caused by the average younger age in the survivor group compared to the non-survivor group.
3. Thrombocytopenia is recognized as a negative prognostic factor in COVID-19 patients. Thrombocytopenia is detected in 5–41.7% of COVID-19 patients (the incidence varies according to disease severity) and it is typically mild. We briefly put it in the Introduction with some literature backgrounds.

# Reviewer report: Reviewer response for version 1

**DOI:** 10.5256/f1000research.120419.r126112

**Published:** 2022-03-14

**Authors:** Martin Westphal

**Affiliations:** 1 University Hospital of Munster, Germany; Fresenius Kabi Deutschland GmbH, Bad Homburg, Germany

This is a very interesting and well conducted retrospective observational study in COVID-19 patients addressing a clinically relevant topic, i.e. the link between D-dimer levels and mortality.

 Please be so kind and clarify the discrepancy in patient number reported in the abstract (259) and the study design (261), which just becomes obvious in the results section. Please clarify also, which data were missing, justifying exclusion. Were these exclusion criteria mentioned *a priori* in the study design/CRF?

 The rather low sensitivity of the D-dimer levels to predict mortality represents a limitation that should be acknowledged and discussed. Are there any means to increase sensitivity, e.g. by combining 2 or more variables?

Is the work clearly and accurately presented and does it cite the current literature?

Yes

If applicable, is the statistical analysis and its interpretation appropriate?

Yes

Are all the source data underlying the results available to ensure full reproducibility?

Yes

Is the study design appropriate and is the work technically sound?

Yes

Are the conclusions drawn adequately supported by the results?

Yes

Are sufficient details of methods and analysis provided to allow replication by others?

Yes

Reviewer Expertise:

Anesthesiology and intensive care

I confirm that I have read this submission and believe that I have an appropriate level of expertise to confirm that it is of an acceptable scientific standard.
